# The Genomics of Isolated Populations of *Gampsocleis glabra* (Orthoptera: Tettigoniidae) in Central and Western Europe

**DOI:** 10.3390/insects14120946

**Published:** 2023-12-14

**Authors:** Oliver Hawlitschek, Carsten Bruns, Lara-Sophie Dey, Soňa Nuhlíčková, Rob Felix, Hein van Kleef, Jacqueline Nakel, Martin Husemann

**Affiliations:** 1 Department of Evolutionary Biology and Environmental Studies, Universität Zürich, 8057 Zürich, Switzerland; 2 Leibniz Institute for the Analysis of Biodiversity Change (LIB), Museum of Nature, 20146 Hamburg, Germany; cbrunsi@web.de (C.B.); lara-sophie.dey@senckenberg.de (L.-S.D.); martin.husemann@smnk.de (M.H.); 3 Senckenberg German Entomological Institute, 15374 Müncheberg, Germany; 4 Department of Ecology, Faculty of Natural Sciences, Comenius University, SK-84215 Bratislava, Slovakia; sona.nuhlickova@uniba.sk; 5 Natuurbalans–Limes Divergens, 6525 ED Nijmegen, The Netherlands; robfelix1@gmail.com; 6 Bargerveen Foundation, 6525 ED Nijmegen, The Netherlands; h.vankleef@science.ru.nl; 7 Department of Environmental and Life Sciences, Biology, Karlstad University, 65188 Karlstad, Sweden; 8 Leibniz Institute of Virology, 20251 Hamburg, Germany; jacqueline.nakel@leibniz-liv.de; 9 Staatliches Museum für Naturkunde Karlsruhe (SMNK), 76133 Karlsruhe, Germany

**Keywords:** bushcricket, conservation, ddRAD, habitat fragmentation, katydid, STRUCTURE

## Abstract

**Simple Summary:**

Habitat destruction is one of the main reasons for the decline and extinction of species. Even if patches of habitat suitable for a certain species persist, the populations of this species may go extinct if the patches are too far apart for individuals to migrate. This is called habitat fragmentation and may also affect species with good migrating abilities if distances are too long. The heath bushcricket *Gampsocleis glabra* is such a species: it is a specialist of steppe-like habitats that are very rare and fragmented across Europe today. We used genetic data to investigate if the remnant populations in Germany are entirely isolated or if individuals are still able to migrate between locations. Our results indicate that all studied populations are isolated to some degree, but a certain degree of gene flow may persist or may have persisted until the recent past. Even today, gene flow appears to persist, possibly with human assistance. This indicates that, aside from the importance of protecting surviving populations in larger habitat fragments, the preservation and restoration of small and isolated patches of steppe-like habitats may be helpful for the conservation of this rare and threatened species.

**Abstract:**

Habitat destruction and fragmentation are among the major current threats to global biodiversity. Fragmentation may also affect species with good dispersal abilities. We study the heath bushcricket *Gampsocleis glabra*, a specialist of steppe-like habitats across Europe that are highly fragmented, investigating if these isolated populations can be distinguished using population genomics and if there are any traces of admixture or dispersal among them. We try to answer these questions using genome-wide SNP data generated with ddRAD sequencing. We calculated F-statistics and visualized differentiation using STRUCTURE plots. While limited by the difficulty of sampling this threatened species, our results show that all populations except one that was represented by a singleton were clearly distinct, with pairwise F_ST_ values between 0.010 and 0.181. STRUCTURE indicated limited but visible admixture across most populations and probably also an exchange of individuals between populations of Germany and The Netherlands. We conclude that in *G. glabra*, a certain amount of gene flow has persisted, at least in the past, also among populations that are isolated today. We also detect a possibly more recent dispersal event between a population in The Netherlands and one in Germany, which may be human aided. We suggest that the conservation of larger populations should be maintained, that efforts should be taken to restore abandoned habitat, that the preservation even of small habitat fragments may be beneficial for the conservation of this species, and that these habitats should be regularly monitored for possible (re-)colonization.

## 1. Introduction

The modification and destruction of natural habitats probably constitutes the greatest threat to global biodiversity [1,2,3]. What further exacerbates this problem is that populations or entire species may go extinct, even while suitable habitats still exist but are fragmented into patches too small and isolated to maintain viable populations [4,5]. With increasing fragmentation of habitats, the quality of individual habitat patches decreases through their small size and potential edge effects, and the chances of complete disappearance increase. The distances between habitat fragments impede the dispersal of organisms. This reduces gene flow and, thereby, the genetic diversity and resilience of a population to environmental change, and it also reduces the chance of re-colonization of a habitat patch after the extinction of a population. Smaller organisms with limited dispersal ability are particularly affected by these phenomena [6]. This includes most terrestrial invertebrates, such as insects. Some flying insect species are highly mobile, but even they will be affected if habitats become small and distances too long [7].

Grasslands, despite covering a substantial amount of our planet’s terrestrial surface, are among the habitat types most threatened by fragmentation. In the Palearctic, steppes still form a nearly continuous belt across the entire temperate zone of Eurasia and neighbouring regions of North Africa and Southwestern Asia. Since their formation around 20 million years ago, these habitats have experienced various cycles of expansion, fragmentation, and decline [8]. Today, human activities are the main drivers of fragmentation and shrinking. Throughout Eurasia, but most conspicuously in Europe, human activities—or the lack thereof—cause degradation of steppes, steppe-like habitats, and other xeric grasslands. Agricultural expansion, intensification, and urbanization fragment and destroy grasslands. However, the abandonment of traditional forms of pastoral land use are equally a threat to these habitats [9]. Furthermore, atmospheric acidifying and the deposition of airborne nitrogen and sulphur has reduced the habitat quality of the remaining grasslands, especially in Northwestern Europe. While large continuous stretches of steppe remain in the Pannonian and Pontic regions of Europe, extrazonal xeric grasslands of Western and Central Europe have been fragmented since the Pleistocene and have particularly steeply declined over the last 100 years [10]. Most of the larger extant patches are situated in protected areas or areas of military use and are maintained by specific management measures [11].

Orthoptera (grasshoppers and bushcrickets) make up a substantial portion of the animal biomass of grassland biomes [12,13]. With around 30,000 known species, Orthoptera are less species-rich than the megadiverse insect orders and, as in most groups, their diversity is higher in tropical than in temperate regions [14]. Nevertheless, the Palearctic steppes are home to a considerable number of species. The global ranges of many of these species follow the distribution of grassland biomes, and they are threatened by the same factors as other biota of these habitats.

One such species is the heath bushcricket (*Gampsocleis glabra* (Herbst, 1786) [15]; Orthoptera: Tettigoniidae), which is among the larger European Orthoptera species with a body length of up to 27 mm [16]. Its global distribution spans from Western Europe to Eastern Asia, but very little is known about the specific localities in the Asian parts of the range. What is known is that populations throughout Europe are fragmented and highly isolated, especially in the Western and Northwestern parts of the range. While the species has been assessed as being of least concern in the IUCN Red List, the population trend is listed as “decreasing” [17]. There are only three populations known in Germany [18,19] and one in The Netherlands [20]. While there are substantial populations in France [21], there is one known population in Poland [22] and two reported in the Czech Republic by Fedor et al. [23]. The species is red-listed and legally protected in all of these countries, except in The Netherlands. *Gampsocleis glabra* is a species of open heathlands and steppe-like habitats and requires a mix of sparse, low vegetation with stands of higher grasses or low shrubs as perches for stridulating males and patches of open soil for oviposition [22,24]. The intensification of agriculture and reforestation measures have caused a steep decline in this type of habitat. Nitrogen deposition and associated acidification have led to grass dominance, moss encroachment and shifts in plant nutrient stoichiometry (overabundance of N in relation to P and other elements [25]) in dry heathlands, the species’ prime habitat in large parts of Western and Central Europe. Plant nutrient stoichiometry is further deteriorated by the abandonment of prescribed burning and the introduction of large-scale sod-cutting [26]. As a result, many extant populations are restricted to protected areas or areas of military use [27]. However, the anatomy of *G. glabra* indicates a well-developed ability to fly and potentially connect or colonize habitats.

Genomic data may elucidate the gene flow between populations of a species and provide insights into the ability of this species to disperse, connect isolated populations, and colonize new habitats. These abilities may be crucial for ensuring the viability of populations and entire species if the species depend on highly specific habitats that are in a fragmented state [28]. Genome-wide data on single-nucleotide polymorphisms (SNPs) has proven useful for such studies because of the high resolution it provides [29]. However, like many species of Orthoptera, the genome of *G. glabra* is most likely very large (>6 Gb) and is assumed to contain vast non-coding stretches, complicating the work with whole genomes [30].

Double-digest restriction-site-associated DNA sequencing (ddRAD) [31] is a method that simplifies the gleaning of genome-wide data by selecting a random subset of the whole genome already in the library preparation step prior to sequencing, while still providing very fine-scale resolution at the population genomic level. This method has the further advantage that genome-wide data can be obtained without the necessity of a reference genome. Despite the anonymous character of the genome fragments sequenced, library preparation is repeatable and complementary datasets can be created if the same set of restriction enzymes is used [32]. ddRAD sequencing has been successfully applied to studies on the genomics of a variety of organisms [33,34,35], including Orthoptera [36,37].

We used ddRAD sequencing on a sample set of *G. glabra* populations from Central and Western Europe to answer the following questions: (1) Can the populations be distinguished using population genomics? And (2) can we find indications of admixture or dispersal among the populations? We then discuss the relevance of our results for conservation management.

## 2. Materials and Methods

**Figure 1:**
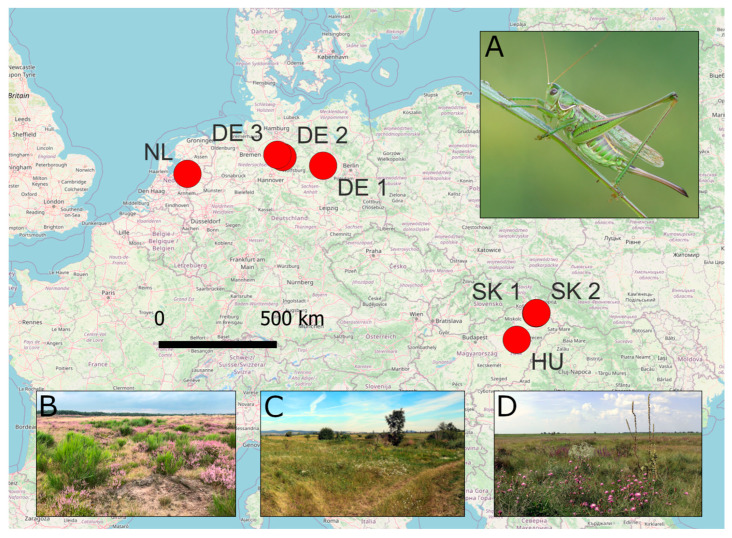
A map of sites sampled in this study. Full names and coordinates are given in Appendix A. Inlays: (**A**): Photograph of *Gampsocleis glabra* by Ján Svetlík. (**B**): Photograph of the heathland habitats of the Oldebroek (NL) site by Hein van Kleef. (**C**): Photograph of the sandy pasture habitats of the Veľký Kamenec (SK 1) site by Oliver Hawlitschek. (**D**): Photograph of the Puszta grassland habitats of the Kócsujfalu (HU) site by Oliver Hawlitschek.

We collected samples of *Gampsocleis glabra* at seven sites in Germany, The Netherlands, Slovakia, and Hungary in July and August 2020, capturing specimens by hand and sweep net (Figure 1, Appendix A). The species is legally protected in Germany, Slovakia, and Hungary; sampling was authorized by the respective local authorities. The sites were selected following recent reports from the last two decades [19,20,24]. We were unable to sample a population in the Colbitz-Letzling Heathlands of Germany [18], but we sampled both other known extant German populations from the Klietz (DE 1) and Lüneburg Heathlands (represented by the Rheinmetall DE 2 and Munster military training areas DE 3). All these sites are heathland habitats that are maintained by specific management measures and are under military use. The selection of sites in Slovakia was based on the reports of Krištín et al. [24]. All sites in Slovakia were xeric grassland habitats on sandy soil with varying degrees of use, mostly as cattle pastures. These sites formed a network of comparatively small patches of a few hectares. The Hungarian site was covered in puszta alkaline grassland vegetation with extensive pastoral use. This site is situated within the Pannonian steppe region, whereas all other sites sampled here are part of extrazonal xeric grasslands.

As the species is rare and generally threatened, we only sampled one hind femur of each specimen. As most Orthoptera readily drop their hind legs when physically threatened, we sampled one hind leg by holding the specimen at the leg after capture with an insect net. The specimens were carefully released after dropping one hind leg, and males resumed singing a few minutes later indicating that the procedure did not cause much stress. We sampled a total of 48 individuals. Genomic DNA was obtained from the hind femora using a high-salt extraction protocol [38].

We separated DNA samples in two pools and prepared libraries for ddRAD sequencing, following the protocol of Peterson [31] with modifications as specified in File S1. We digested each 1000 ng of DNA per sample overnight using the restriction enzymes SbfI and MseI in reactions of 50 μL. We then ligated Illumina P1 and P2 adapters with individual 5-bp barcodes, equally in reactions of 50 μL for three hours and checked the fragment length distribution using the Agilent TapeStation electrophoresis platform (Agilent Technologies, Inc., Santa Clara, CA, USA). After that, samples were pooled with a Qiagen MinElute PCR Purification Kit (Qiagen N.V., Hilden, Germany), and size-selection of DNA fragments to 300 bp was conducted with BluePippin. Subsequently, we amplified the size-selected pool in ten separate PCR reactions with 18 cycles, and another pooling and size-selection step was conducted. All purification steps, except the pooling step, were conducted with magnetic beads. The final pools of both libraries were sequenced separately, paired-end 2 × 150 bp on an Illumina NextSeq 500 platform (Illumina, San Diego, CA, USA) of the Leibniz-Institute of Virology (LIV). The first library was sequenced with the addition of 1% PHIX, and the second with 10%.

We used FastQC (v0.11.9; https://www.bioinformatics.babraham.ac.uk/projects/fastqc/, accessed on 1 November 2023) to assess the quality of the raw reads and then demultiplexed the data using the *process_radtags* component of the STACKS pipeline [39,40] at the computing cluster of LIB, trimming the length of all sequences to 75 bp. After further trimming the initial ten base pairs of every read with Cutadapt [41], we continued processing the results in STACKS with a priori assignment of samples to populations according to their localities. We ran the final *population* process with r = 0.5, keeping only the first SNP per locus with the option –write-single-snp.

F-statistics were calculated in the adegenet package [42,43] in R [44]. We estimated the proportional genetic variance F_ST_ and the inbreeding coefficient F_IS_ as total, F_IS_ for all a priori defined populations, and F_ST_ of all pairwise comparisons. Further downstream analyses were conducted in the STRUCTURE v2.3.4 software [45]. We analysed the dataset for the span of K = 2 to K = 10 in two iterations for 100,000 generations each with a burn-in of 10,000. Using the method of Evanno [46] in STRUCTURE HARVESTER v0.6.94 [47], we calculated ΔK to select the optimal K for our results. We visualized STRUCTURE results in CLUMPAK [48].

## 3. Results

We detected the target species at all surveyed sites in Germany, The Netherlands, and Hungary. In Slovakia, we detected *Gampsocleis glabra* at two sites. We also surveyed four further sites where Krištín et al. [24] detected the species in 2005–2006 but failed to find any individuals there.

Quality checks and initial raw data processing using FastQC and the *process_radtags* component of STACKS yielded a total of 345,007,878 raw reads for the first library (1% PHIX) and 251,915,490 for the second (10% PHIX). However, *process_radtags* failed to find barcodes in ca. 50% of all raw reads of the first library, but only in <1% of the second, resulting in the retention of 157,601,622 and 230,084,411 reads for the two libraries, respectively. In all further steps, after eliminating poor-quality reads and samples, the reads from both libraries were analyzed together. The dataset generated by the *population* process of STACKS consisted of 37 out of the originally 48 individuals from all seven sampled populations (Table 1). STACKS retrieved 87,832 loci with 21,233 variant sites.

Overall proportional genetic variance F_ST_ was calculated as 0.049, and overall inbreeding F_IS_ as 0.048. Pairwise F_ST_ (pairwise genetic divergence) and F_IS_ per individual population are given in Table 2. Pairwise F_ST_ values ranged from 0.010 (populations Klietz − DE 1/Rheinmetall − DE 2) to 0.181 (Oldebroek − NL/Munster − DE 3). F_IS_ per population was found to be lowest in Kócsujfalu (HU), with 0.184 and highest in Klietz (DE 1), with 0.600.

**Figure 2:**
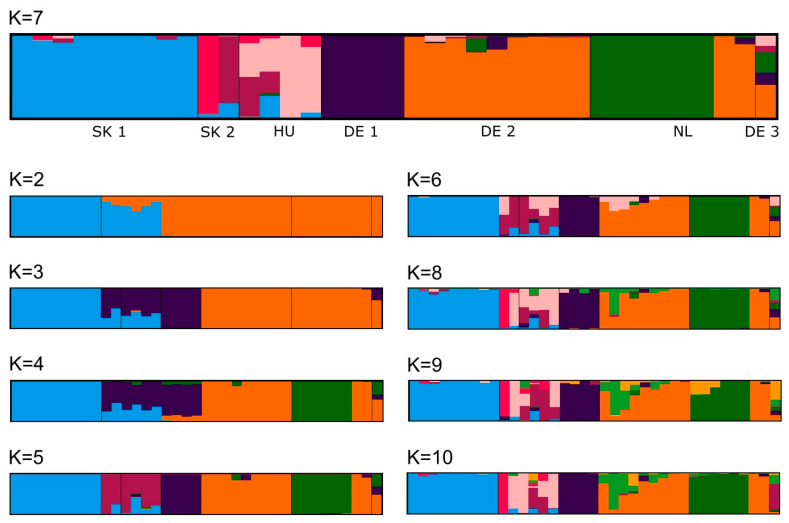
Plots generated in STRUCTURE. The plot of K = 7 is shown enlarged on top because this K was favoured by the Evanno analysis. Population codes are given in Table 1.

The STRUCTURE plots are given in Figure 2. STRUCTURE HARVESTER detected the greatest K (102.09) in K = 7. In K = 7, the populations SK 1, DE 1, and DE 2 are largely represented by unique genotypes that are only found as slight traces of admixture in other populations. The populations SK 2 and HU each have a unique genotype plus an additional shared genotype. NL also has a unique genotype, but there are two individuals from this population that were assigned to the genotype from DE 2. The single individual from DE 3 was retrieved as a mixture of genotypes from other populations.

Notably, in K = 2, STRUCTURE distinguishes between populations from Slovakia and Hungary versus Germany and The Netherlands. SK 2, HU, and DE 1 were found to share a genotype in K = 3 and K = 4, only SK 2 and HU in K = 5. The distinct genotype from NL was retrieved from K = 4 through to K = 10, with the individuals sharing the genotype from DE 2 always visible.

## 4. Discussion

We studied the population genomics of one of the rarest bushcrickets of Western and Central Europe, *Gampsocleis glabra*. Our results suggest substantial isolation with limited gene flow, especially among the Dutch and German populations, but also in the Eastern part of the studied range.

We did not measure population densities of *G. glabra*, but we found the effort required to collect samples varying across sites. In combination with our efforts to keep the disturbance of this threatened species at a minimum, this resulted in comparatively low and inhomogeneous sample sizes across populations. This structure of our sampling certainly distorts the analyses and mandates specific caution while interpreting the results. In particular, the Munster (DE 3) site is represented by a single individual, largely excluding it from analytical study. Strážne (SK 2) is represented by only two samples. Nevertheless, we believe that some insights can be gained from our results, especially as DE 2 and DE 3 probably represent connected subpopulations from the Lüneburg area.

The results of pairwise F_ST_ were probably distorted by the inclusion of the single sample from Munster (DE 3). Excluding this population, pairwise F_ST_ ranges from 0.010–0.139, clearly distinguishing the populations. Studies on other Orthoptera sometimes found substantially higher F_ST_ values in wild populations, such as 0.235–0.357 per gene in *Pseudochorthippus parallelus* (Acrididae, [49,50]). Other studies found wider ranges, as demonstrated for *Isophya* bushcrickets (Tettigoniidae) with −0.007–0.173 [51]/−0.052–0.412 [52] for *I. kraussi* and −0.056–0.593 for *I. stysi* [52] (N = 10 populations in both studies). On the other hand, Xu et al. [53] detected F_ST_ of a similar, albeit slightly lower range as found by us in *Calliptamus italicus* (Acrididae) with −0.009–0.125, while Nolen et al. [54] retrieved even lower maximum values of 0.057–0.105 between separate species of the *Chorthippus biguttulus* group. This comparison indicates that the populations of *G. glabra*, a species with good flying and dispersal ability, studied by us have not been isolated any more than other species of Orthoptera that had previously been studied. This can at least be assumed for a historically more interconnected population network. The values found by us are lower than those of *Pseudochorthippus* and *Isophya*, which have limited or no capability of flight (despite the occurrence of macropterous males in *Pseudochorthippus*) and are more comparable to strong fliers such as *Calliptamus* or *Chorthippus*.

The inbreeding coefficient F_IS_ is 0.184 or larger for all populations, suggesting a deficit of heterozygotes. The lowest value was found in the population from Kócsujfalu (HU), which inhabits a large area of suitable habitat and is probably already part of a larger continuous metapopulation of the Pannonian steppes [55]. While we do not have the data to calculate effective population size, the large area suggests a comparatively big population. All other sampled populations probably inhabit smaller habitat fragments and also have higher inbreeding coefficients. German and Dutch sites are situated in areas with military use, probably resulting in frequent bottlenecks even in larger populations after diebacks through fire from military activity or prescribed burning. For the Hungarian–Slovakian area, our results suggest a scenario in which the population that we sampled at Kócsujfalu acts as a source, whereas the Veľký Kamenec (SK 1) population with F_IS_ = 0.523 probably represents a sink, and Strážne (SK 2, F_IS_ = 0.235) an intermediate, even though SK 1 and SK 2 are geographically much closer to the sampling locality in HU. In Germany and The Netherlands, F_IS_ values are overall lower. Notably, the highest inbreeding (F_IS_ = 0.600) was found in the Klietz (DE 2) populations, which Schäfer & Hennings [19] reported as the most likely largest populations of (Western?) Central Europe. This suggests that these geographically more remote populations, despite their substantial size, may suffer from genetic deprivation through inbreeding caused by the long geographic distances of these sinks to any source population.

Notably, studies of other Orthoptera also found largely positive F_IS_, such as 0.311–0.437 in *I. kraussi* and 0.293–0.333 in *I. stysi* [52]. As these are, unlike *G. glabra*, flightless species, even geographically closer populations are likely more isolated.

The STRUCTURE plots show many distinct genotypes and many populations with little, but nevertheless visible admixture. In K = 7, which was favoured by the Evanno analysis and matched the a priori designation of populations, all populations are distinct and represented by a unique genotype; except Munster (DE 3), which is probably an artefact caused by the singleton. On the other hand, a certain degree of admixture is visible in all populations except Klietz (DE 1). There is a specific genotype shared between the populations of Strážne (SK 2) and Kócsujfalu (HU), and both populations also show admixture from Veľký Kamenec (SK 1). Despite the proximity of SK 1 and SK 2, there may be more gene flow between SK 2 and HU. However, the comparatively lower sample size of SK 2 (N = 2) vs. SK 1 (N = 9) and HU (N = 4) may distort the results to a certain degree, which should therefore be treated with caution. The population from Oldebroek (NL) consists both of individuals with a genotype assigned almost uniquely to this site and of individuals assigned to the genotype from Rheinmetall (DE 2), >250 km apart without any known populations in between. This absence of admixture suggests a relatively recent dispersal event. While active migration over such long distances cannot be ruled out, it seems very unlikely. If natural migration out of the NL population occurred, at least rare sightings of *G. glabra* in nearby heathlands could be assumed, but no such observations are known (H.v.K. and R.F. pers. obs.). Instead, the dispersal may have been supported by human activity, possibly in the framework of Dutch–German military collaboration.

Overall, our results highlight two main insights into the populations of *G. glabra*: (1) The widespread admixture indicates that populations of extrazonal xeric grasslands, at least in Germany and The Netherlands, have at least historically been connected through gene flow that must have been mediated by active dispersal of individuals. Possibly, a network of stepping stones existed that facilitated migration among larger populations. However, while the number of known populations in Central and Western Europe was historically somewhat higher, Harz [56] reports only “very sparse occurrences” already in 1957, suggesting that many of the extant populations have been isolated for most of the 20th century at least. Since we have no specific dating for the results of our population genomic analyses, we cannot determine the age of the admixture we observe. However, the relatively common admixture found in many populations suggests that the active migration of individuals occurs at least occasionally. (2) Genotypes may be shared between relatively distant populations, as exemplified in Oldebroek (NL) and Rheinmetall (DE 2). This sharing of genotypes indicates recent dispersal. While active migration cannot be ruled out, incidental or planned human translocation may have also played a role. Our data do not allow any assessments of how much of this migration is currently happening naturally vs. under human influence, intentionally or unintentionally.

Our surveys found that *G. glabra* most likely disappeared from sites at which populations existed just a few decades ago [24], which is probably due to the modification of the habitats. On the other hand, the admixture we found suggests that this species can probably maintain a certain degree of gene flow if populations are geographically separate to some degree. This, in turn, indicates a certain dispersal capacity. In this context, larger source populations may have maintained sink populations in smaller habitat patches, which also served as stepping stones for the migration of individuals between the larger populations. Today, most populations we studied are probably too far apart to allow for active migration of individuals and thus maintain gene flow. Nevertheless, some dispersal still seems possible across distances. A previously unknown population of *G. glabra* was recently found in a very small area near Munich, Germany (J. Brozio and J. Voith, pers. comm. July 2022). The meadow habitat was established only in the 1990 years in the course of the restoration of the area of the former airport of Munich-Riem. There are no historical records of *G. glabra* in that area; the closest historically known populations, both extinct by the 1950s, were at distances of about 20 km (Garchinger Heide) and 60 km (Königsbrucker Heide) [57]. Both historical localities have been intensely surveyed since. It is unknown whether the new population originated from the active migration of individuals or from human-aided dispersal. In any case, this event suggests that—no matter whether the site was colonized naturally or through human translocation—the species is able to establish populations in limited patches of suitable habitat.

## 5. Conclusions

Our analyses found admixture between many populations of *Gampsocleis glabra*, which may be the result of historical connectivity between populations in extrazonal grassland habitats, but also the sharing of genotypes between populations, which may be the result of more recent—possibly human-aided—dispersal. Therefore, we recommend three parts of a strategy for the conservation management of *G. glabra* in extrazonal grassland habitats: (1) Management strategies for the habitats of existing larger populations should be maintained, as is already being carried out. (2) Small populations of *G. glabra*, or even small patches of habitat that appear suitable or have been inhabited before, are worth preserving as parts of a habitat network that may sustain the metapopulation. In the long term, however, the survival of populations with a lower genetic variability, i.e., possible sinks, can only be ensured through connectivity with larger source populations. (3) Sites with suitable habitats and sites that once harboured populations should be monitored regularly for possible re-colonization, and restoration options should be explored for these sites.

## Figures and Tables

**Table 1:** List of samples of *Gampsocleis glabra* used in this study. The locality column provides population codes used in Figure 2 and Table 2.

No.	Sex	Collection Date	Collector	Country	Locality
ML20	f	13 August 2020	OH	Slovakia	Veľký Kamenec (SK 1)
ML21	f	13 August 2020	OH	Slovakia	Veľký Kamenec (SK 1)
ML23	m	13 August 2020	OH	Slovakia	Veľký Kamenec (SK 1)
ML26	f	13 August 2020	OH	Slovakia	Veľký Kamenec (SK 1)
ML27	f	13 August 2020	OH	Slovakia	Veľký Kamenec (SK 1)
ML29	f	13 August 2020	OH	Slovakia	Veľký Kamenec (SK 1)
ML30	m	13 August 2020	OH	Slovakia	Strážne (SK 2)
ML32	m	13 August 2020	OH	Hungary	Kócsujfalu (HU)
ML33	m	13 August 2020	OH	Hungary	Kócsujfalu (HU)
ML34	f	13 August 2020	OH	Hungary	Kócsujfalu (HU)
ML35	m	13 August 2020	OH	Hungary	Kócsujfalu (HU)
ML52	m	17 August 2020	MH	Germany	Klietz (DE 1)
ML54	m	17 August 2020	MH	Germany	Klietz (DE 1)
ML58	m	17 August 2020	MH	Germany	Klietz (DE 1)
ML62	m	17 August 2020	MH	Germany	Klietz (DE 1)
ML70	f	28 August 2020	MH	Germany	Rheinmetall (DE 2)
ML71	f	28 August 2020	MH	Germany	Rheinmetall (DE 2)
ML73	f	28 August 2020	MH	Germany	Rheinmetall (DE 2)
ML75	m	28 August 2020	MH	Germany	Rheinmetall (DE 2)
ML76	m	28 August 2020	MH	Germany	Rheinmetall (DE 2)
ML77	m	28 August 2020	MH	Germany	Rheinmetall (DE 2)
ML88	m	31 July 2020	RF, HvK	The Netherlands	Oldebroek (NL)
ML89	m	31 July 2020	RF, HvK	The Netherlands	Oldebroek (NL)
ML90	m	31 July 2020	RF, HvK	The Netherlands	Oldebroek (NL)
ML92	m	31 July 2020	RF, HvK	The Netherlands	Oldebroek (NL)
ML93	m	31 July 2020	RF, HvK	The Netherlands	Oldebroek (NL)
ML94	m	31 July 2020	RF, HvK	The Netherlands	Oldebroek (NL)
ML99	m	13 August 2020	OH	Slovakia	Strážne (SK 2)
ML100	f	13 August 2020	OH	Slovakia	Veľký Kamenec (SK 1)
ML101	m	13 August 2020	OH	Slovakia	Veľký Kamenec (SK 1)
ML102	f	13 August 2020	OH	Slovakia	Veľký Kamenec (SK 1)
ML104	m	27 August 2020	MH	Germany	Munster (DE 3)
ML109	m	28 August 2020	MH	Germany	Rheinmetall (DE 2)
ML110	m	28 August 2020	MH	Germany	Rheinmetall (DE 2)
ML112	m	28 August 2020	MH	Germany	Rheinmetall (DE 2)
ML114	m	28 August 2020	MH	Germany	Rheinmetall (DE 2)
ML115	m	28 August 2020	MH	Germany	Rheinmetall (DE 2)

**Table 2:** Pairwise F_ST_ and F_IS_ per population (Pop.). Population codes are given in Table 1.

Pop.	SK 1	SK 2	HU	DE 1	DE 2	NL	DE 3
N	9	2	4	4	11	6	1
SK 2	0.036						
HU	0.139	0.106					
DE 1	0.124	0.086	0.090				
DE 2	0.115	0.081	0.081	0.037			
NL	0.010	0.031	0.139	0.121	0.108		
DE 3	0.165	0.068	0.147	0.038	0.051	0.181	
F_IS_	0.523	0.235	0.184	0.600	0.463	0.282	0.000

## Data Availability

The raw sequencing reads are available under submission SUB14031511 at the NCBI sequencing reads archive (SRA).

## Supplementary Materials

The following supporting information can be downloaded at: https://www.mdpi.com/article/10.3390/insects14120946/s1, File S1: A protocol for the ddRAD library preparation adapted for this study.

## Appendix A

**Table A1:** Geographic coordinates (in WGS84) and description of the sites sampled in this study.

Locality	Code	Latitude	Longitude	Description
Veľký Kamenec	SK 1	48.3741	21.8254	Sand dunes with high grass
Strážne	SK 2	48.3875	21.8465	Cow pasture with high grass
Kócsujfalu	HU	47.5703	20.9444	Puszta meadows, pasture
Klietz	DE 1	52.6451	12.1318	*Calluna* heathland, military training
Rheinmetall	DE 2	52.8829	10.2861	*Calluna* heathland, military training
Oldebroek	NL	52.4194	5.9575	*Calluna* heathland, military training
Munster	DE 3	52.9451	10.0502	*Calluna* heathland, military training

## References

[1] Pereira H.M., Leadley P.W., Proença V., Alkemade R., Scharlemann J.P.W., Fernandez-Manjarrés J.F., Araújo M.B., Balvanera P., Biggs R., Cheung W.W.L. (2010). Scenarios for Global Biodiversity in the 21st Century. Science (1979).

[2] Pardini R., Nichols E., Püttker T. (2017). Biodiversity Response to Habitat Loss and Fragmentation. Encycl. Anthr..

[3] Hoekstra J.M., Boucher T.M., Ricketts T.H., Roberts C. (2005). Confronting a Biome Crisis: Global Disparities of Habitat Loss and Protection. Ecol. Lett..

[4] Wilson M.C., Chen X.Y., Corlett R.T., Didham R.K., Ding P., Holt R.D., Holyoak M., Hu G., Hughes A.C., Jiang L. (2016). Habitat Fragmentation and Biodiversity Conservation: Key Findings and Future Challenges. Landsc. Ecol..

[5] Fahrig L. (2003). Effects of Habitat Fragmentation on Biodiversity. Annu. Rev. Ecol. Evol. Syst..

[6] Louy D., Habel J.C., Schmitt T., Assmann T., Meyer M., Müller P. (2007). Strongly Diverging Population Genetic Patterns of Three Skipper Species: The Role of Habitat Fragmentation and Dispersal Ability. Conserv. Genet..

[7] Dey L.S., Simões M.V.P., Hawlitschek O., Sergeev M.G., Xu S.Q., Lkhagvasuren D., Husemann M. (2021). Analysis of Geographic Centrality and Genetic Diversity in the Declining Grasshopper Species *Bryodemella tuberculata* (Orthoptera: Oedipodinae). Biodivers. Conserv..

[8] Gibson D.J. (2009). Grasses and Grassland Ecology.

[9] Török P., Neuffer B., Heilmeier H., Bernhardt K.G., Wesche K. (2020). Climate, Landscape History and Management Drive Eurasian Steppe Biodiversity. Flora.

[10] Kajtoch Ł., Cieślak E., Varga Z., Paul W., Mazur M.A., Sramkó G., Kubisz D. (2016). Phylogeographic Patterns of Steppe Species in Eastern Central Europe: A Review and the Implications for Conservation. Biodivers. Conserv..

[11] Reif J., Chajma P., Dvořáková L., Koptík J., Marhoul P., Čížek O., Kadlec T. (2023). Biodiversity Changes in Abandoned Military Training Areas: Relationships to Different Management Approaches in Multiple Taxa. Front. Environ. Sci..

[12] Porter E.E., Redak R.A., Braker H.E. (1996). Density, Biomass, and Diversity of Grasshoppers (Orthoptera: Acrididae) in a California Native Grassland. Great Basin Nat..

[13] Branson D.H., Joern A., Sword G.A. (2006). Sustainable Management of Insect Herbivores in Grassland Ecosystems: New Perspectives in Grasshopper Control. Bioscience.

[14] Cigliano M.M., Braun H., Eades D.C., Otte D. Orthoptera Species File Version 5.0/5.0. https://orthoptera.speciesfile.org/.

[15] Herbst J.W., Fuessly J.C. (1786). Forsetzung Des Verzeichnisses Meiner Insektensammlung. Archiv Der Insectengeschichte.

[16] Bellmann H., Rutschmann F., Roesti C., Hochkirch A. (2019). Der Kosmos Heuschrecken-Führer.

[17] Hochkirch A. *Gampsocleis glabra* (Steppe Spiny Bush-Cricket). https://www.iucnredlist.org/species/44711951/115472224.

[18] Schäfer B. (2013). Nachweis Der Heideschrecke *Gampsocleis glabra* (Herbst, 1786) (Ensifera) in Der Colbitz-Letzlinger Heide (Sachsen-Anhalt). Articulata.

[19] Schäfer B., Hennigs S. (2020). Nachweise Der Heideschrecke *Gampsocleis glabra* (Herbst, 1786) (Ensifera) in Der Altengrabower Sowie in Der Klietzer Heide (Brandenburg/Sachsen-Anhalt). Articulata.

[20] van der Berg A., Haveman R., Hornman M. (2000). De Kleine Wrattenbijter *Gampsocleis glabra* Herontdekt in Nederland (Orthoptera: Tettigoniidae). Ned. Faun. Meded..

[21] Sardet E., Roesti C., Braud Y. (2015). Cahier d’identification Des Orthoptères de France, Belgique, Luxembourg & Suisse.

[22] Grzędzicka E., Vahed K. (2020). Habitat Requirements of the Endangered Heath Bush-Cricket *Gampsocleis glabra* (Orthoptera, Tettigoniidae) in an Isolated Population. J. Insect Conserv..

[23] Fedor P., Holuša J., Majzlan O., Prokop P. (2004). Distribution, Conservation and Prognosis for *Gampsocleis glabra* (Herbst, 1786) (Insecta: Ensifera) in Slovakia and the Czech Republic. Articulata.

[24] Krištín A., Kaňuch P., Balla M., Gavlas V. (2007). On the Distribution and Ecology of *Gampsocleis glabra* and *Tettigonia caudata* (Orthoptera) in Slovakia. Articulata.

[25] Vogels J.J., Van de Waal D.B., Wallis de Vries M.F., Van den Burg A.B., Nijssen M., Bobbink R., Berg M.P., Olde Venterink H., Siepel H. (2023). Towards a Mechanistic Understanding of the Impacts of Nitrogen Deposition on Producer–Consumer Interactions. Biol. Rev..

[26] Vogels J.J., Verberk W.C.E.P., Lamers L.P.M., Siepel H. (2017). Can Changes in Soil Biochemistry and Plant Stoichiometry Explain Loss of Animal Diversity of Heathlands?. Biol. Conserv..

[27] Roesti C., Rutschmann F. Orthoptera.Ch. http://www.orthoptera.ch/.

[28] Pierson J.C., Beissinger S.R., Bragg J.G., Coates D.J., Gerard J., Oostermeijer B., Sunnucks P., Schumaker N.H., Trotter M.V., Young A.G. (2015). Incorporating Evolutionary Processes into Population Viability Models. Conserv. Biol..

[29] Formenti G., Theissinger K., Fernandes C., Bista I., Bombarely A., Bleidorn C., Ciofi C., Crottini A., Godoy J.A., Höglund J. (2022). The Era of Reference Genomes in Conservation Genomics. Trends Ecol. Evol..

[30] Hawlitschek O., Sadílek D., Dey L.S., Buchholz K., Noori S., Baez I.L., Wehrt T., Brozio J., Trávníček P., Seidel M. (2023). New Estimates of Genome Size in Orthoptera and Their Evolutionary Implications. PLoS ONE.

[31] Peterson B.K., Weber J.N., Kay E.H., Fisher H.S., Hoekstra H.E. (2012). Double Digest RADseq: An Inexpensive Method for de Novo SNP Discovery and Genotyping in Model and Non-Model Species. PLoS ONE.

[32] Escoda L., Hawlitschek O., González-Esteban J., Castresana J. (2022). Methodological Challenges in the Genomic Analysis of an Endangered Mammal Population with Low Genetic Diversity. Sci. Rep..

[33] Çilingir F.G., Hansen D., Ozgul A., Grossen C. (2021). Design of SNP Markers for Aldabra Giant Tortoises Using Low Coverage DdRAD-Seq. Conserv. Genet. Resour..

[34] Roy S.C., Moitra K., De Sarker D. (2017). Assessment of Genetic Diversity among Four Orchids Based on DdRAD Sequencing Data for Conservation Purposes. Physiol. Mol. Biol. Plants.

[35] Querejeta M., González-Esteban J., Gómez A., Fernández-González A., Aymerich P., Gosálbez J., Escoda L., Igea J., Castresana J. (2016). Genomic Diversity and Geographical Structure of the Pyrenean Desman. Conserv. Genet..

[36] Noguerales V., Ortego J. (2022). Genomic Evidence of Speciation by Fusion in a Recent Radiation of Grasshoppers. Evolution.

[37] González-Serna M.J., Cordero P.J., Ortego J. (2018). Using High-Throughput Sequencing to Investigate the Factors Structuring Genomic Variation of a Mediterranean Grasshopper of Great Conservation Concern. Sci. Rep..

[38] Paxton R.J., Thorén P.A., Tengö J., Estoup A., Pamilo P. (1996). Mating Structure and Nestmate Relatedness in a Communal Bee, Andrena Jacobi (Hymenoptera, Andrenidae), Using Microsatellites. Mol. Ecol..

[39] Catchen J., Hohenlohe P.A., Bassham S., Amores A., Cresko W.A. (2013). Stacks: An Analysis Tool Set for Population Genomics. Mol. Ecol..

[40] Catchen J.M., Amores A., Hohenlohe P., Cresko W., Postlethwait J.H. (2011). Stacks: Building and Genotyping Loci de Novo from Short-Read Sequences. G3 Genes Genomes Genet..

[41] Martin M. (2011). Cutadapt Removes Adapter Sequences from High-Throughput Sequencing Reads. EMBnet J..

[42] Jombart T. (2008). Adegenet: A R Package for the Multivariate Analysis of Genetic Markers. Bioinformatics.

[43] Jombart T., Ahmed I. (2011). Adegenet 1.3-1: New Tools for the Analysis of Genome-Wide SNP Data. Bioinformatics.

[44] R Core Team (2020). R: A Language and Environment for Statistical Computing.

[45] Pritchard J.K., Stephens M., Donnelly P. (2000). Inference of Population Structure Using Multilocus Genotype Data. Genetics.

[46] Evanno G., Regnaut S., Goudet J. (2005). Detecting the Number of Clusters of Individuals Using the Software STRUCTURE: A Simulation Study. Mol. Ecol..

[47] Earl D.A., von Holdt B.M. (2012). Structure Harvester: A Website and Program for Visualizing STRUCTURE Output and Implementing the Evanno Method. Conserv. Genet. Resour..

[48] Kopelman N.M., Mayzel J., Jakobsson M., Rosenberg N.A., Mayrose I. (2015). Clumpak: A Program for Identifying Clustering Modes and Packaging Population Structure Inferences across K. Mol. Ecol. Resour..

[49] Hagberg L., Celemín E., Irisarri I., Hawlitschek O., Bella J.L., Mott T., Pereira R.J., Ricardo Pereira C.J. (2022). Extensive Introgression at Late Stages of Species Formation: Insights from Grasshopper Hybrid Zones. Mol. Ecol..

[50] Korkmaz E.M., Sari M., Başibüyük H.H. (2010). Genetic Structure of *Chorthippus parallelus* (Orthoptera: Acrididae: Gomphocerinae) Populations in Anatolia: A Stable Rear Edge Population. Ann. Entomol. Soc. Am..

[51] Pecsenye K., Vadkerti E., Academiae Z.V.-A.Z. (2003). Temporal and Spatial Pattern of Genetic Differentiation in *Isophya kraussii* (Orthoptera: Tettigonoidea) in NE Hungary. Acta Zool. Acad. Sci. Hung..

[52] Pecsenye K., Vadkerti E., Varga Z. (2003). Pattern of Genetic Differentiation in Two *Isophya* Species (Orthoptera: Tettigonoidea) in North-East Hungary. J. Insect Conserv..

[53] Xu Y., Mai J., Yu B., Hu H., Yuan L., Jashenko R., Ji R. (2019). Study on the Genetic Differentiation of Geographic Populations of *Calliptamus italicus* (Orthoptera: Acrididae) in Sino-Kazakh Border Areas Based on Mitochondrial COI. J. Econ. Entomol..

[54] Nolen Z.J., Yildirim B., Irisarri I., Liu S., Groot Crego C., Amby D.B., Mayer F., Gilbert M.T.P., Pereira R.J. (2020). Historical Isolation Facilitates Species Radiation by Sexual Selection: Insights from *Chorthippus* Grasshoppers. Mol. Ecol..

[55] Kelemen A., Török P., Valkó O., Deák B., Miglécz T., Tóth K., Ölvedi T., Tóthmérész B. (2014). Sustaining Recovered Grasslands Is Not Likely without Proper Management: Vegetation Changes after Cessation of Mowing. Biodivers. Conserv..

[56] Harz K., Fischer G. (1957). Die Geradflügler Mitteleuropas.

[57] Schlumprecht H., Waeber G. (2003). Heuschrecken in Bayern.

