# Supplementary material for: The Genomics of Isolated Populations of Gampsocleis glabra (Orthoptera: Tettigoniidae) in Central and Western Europe
Source: Insects. 2023 Dec 14;14(12):946. doi: 10.3390/insects14120946 (PMC10743875; doi:10.3390/insects14120946)
Supplement: Supplementary file 1 [file insects-14-00946-s001.zip › insects-2698989-supplementary.pdf]

File S1: A protocol for the ddRAD library preparation adapted for this study.
Carsten Bruns
14 June 2022

Adapter ligation

- Prepare adapter mix for each adapter:
(best place in 8 strips for easy pipetting)
- 1 µl Adapter part 1
- 1 µl Adapter part 2
- 8 µl AB Buffer

- PCR:
98°C (2.5 min)
Cool down by 3°C/min to 21°C
Hold at 4°C

Digestion

- Adapters should be prepared by annealing not too long before digestion.

- Measure DNA concentration of the samples in Nanodrop or Qbit. DNA concentration of samples should be between 50 and 100 ng/µl.

- A total of 300 – 1200 ng of DNA should be used. This is achieved by diluting and using 1 – 25 µl of every sample for the digestion.

- Prepare digestion mix (add water / adapter first, then buffer, enzymes last; cool). Per sample:
- 5 µl Buffer CutSmart 10x
- 1 µl SbfI (TS)
- 1 µl MseI (TS)

- To every tube, add:
- 7 µl mix
- 1 - 25 µl of DNA sample
- 14 - 8 µl of H_2O
(-> 50 µl total)

- Incubate at 37 °C for 15 minutes.

- Inactivate enzymes at 80 °C for 20 minutes.

- Purify using magnetic beads. Spin down.

- Check fragment lengths, e.g., with Tapestation.

Enzymes used here:
MseI: 10 000 U/mL, 2500 U, NEB R0525L
SbfI: 10 000 U/mL, 2500 U, NEB R0642L
The volumes of enzymes to be used may vary by manufacturer. The protocol must be adapted accordingly.

Ligation

- Prepare ligation mix (add water / adapter first, then buffer, enzymes last; cool). Per sample:
- 8 µl H_2O MQ
- 2 µl Adapter P2, diluted
- 5 µl Buffer T4 10X
- 4 µl Ligase T4 (TS; 5 u/µl)

- To every tube, add:
- 18 µl ligation mix
- 2 µl individual Adapter P1, diluted, with corresponding barcode
- 30 µl digested DNA

(-> 50 µl total)

- Incubate at room temperature, at least ~2 h or overnight.

- Inactivate by heating to 65 ºC, 10 min. Cool down to room temperature. Spin down.

- Check fragment lengths, e.g., with Tapestation.

Enzymes used here:
Ligase T4: 400 000 U/mL, 100 000 U, NEB M0202L
The volumes of enzymes to be used may vary by manufacturer. The protocol must be adapted accordingly.

Pool / Size-selection 1

- Add 250 µl of buffer PB to every tube of 50 µl of ligation product (to finalize inactivation of ligase).

- Pool all samples in one tube (Falcon 15 ml sterile).

- Run the whole of the pooled samples through 1 column of MinElute PCR Purification Kit in steps of ~750 µl; centrifuge each time after adding volume.

- Elute in 30 µl of EB, incubate 5 min.

Double-sided size selection with beads:

- Add beads at x0.45 volume; mix
- Incubate 5 min at RT, then place on magnetic rack [or plate] for 2 min
- Transfer supernatant and to new PCR tube
- Add beads at x0.35 volume; mix
- Incubate 5 min at RT, then place on magnetic rack for 2 min
- Discard supernatant
- Add 200 µL of 70% EtOH and incubate 30 s
- Discard EtOH
- Repeat EtOH washing
- Dry for 10 min
- Remove sample from magnetic rack and add 30 µL EB, pipette up & down 10 times
- Incubate 2 min
- Place on magnetic rack for 1 min
- Transfer supernatant to new PCR tube
- Check DNA concentration in Qubit [wait at least 1 hour after size selection]

- Check fragment lengths, e.g., with Tapestation.

PCR

- Prepare a mix (per sample):
- 13.25 µl H2O MQ
- 1 µl Primer PCR1 (2 µM final)
- 1 µl Primer PCR2 (2 µM final)
- 5 µl PHUSION buffer HF 5x
- 0.5 µl 10 mM dNTPs
- 0.25 µl PHUSION Polymerase

- Join:
- 21 µl mix
- 4 µl size-selected sample

- PCR:
Initial denaturation: 98 ºC (30 sec)
Cycles: 98 ºC (10 sec); 65 ºC (30 sec); 72 ºC (30 sec)
Final extension: 72 ºC (5 min)
18 cycles

- Run 5 µl of sample in 1% agarose gel. The library must be ca. 50 bp longer than the selected size (= 350 - 450 bp).

- Do 3 – 6 PCRs, if possible, with fewer cycles, if necessary with more (14 – 20).

Pool / Size selection 2

- Pool all PCR products: 20 µl each. Concentrate with MinElute PCR Purification Kit (20 µl of EB, 5 min incubation).

- Measure final elution with Nanodrop (1.5 µl). DNA concentration must be between 1 – 20 ng/µl.

- Run size selection with Blue Pippin (to 300 bp).
